# Tuning hydrogel properties and Schwann cell behavior through microchannel size control in magnetically templated hydrogels

**DOI:** 10.1039/d5bm01573a

**Published:** 2026-04-30

**Authors:** Victor G. Rivera-Llabres, Zoe A. Fields, Hayden J. Good, Elizabeth L. Aikman, Stephanie Manrique, Adeline Schmidt, Eleana Manousiouthakis, Whitney L. Stoppel, Christine E. Schmidt, Carlos M. Rinaldi-Ramos

**Affiliations:** a Department of Chemical Engineering, University of Florida Gainesville FL 32611 USA carlos.rinaldi@ufl.edu; b Department of Agricultural and Biological Engineering, University of Florida Gainesville FL 32611 USA; c J. Crayton Pruitt Department of Biomedical Engineering, University of Florida Gainesville FL 32611 USA

## Abstract

Peripheral nerve injury is a common condition and the development of materials that enhance Schwann cell migration to aid in regeneration is critical as the body has a limited ability to repair transected nerves. While various techniques have introduced 2D and 3D physical cues in biomaterials to promote Schwann cell infiltration, developing cell-scale tubular channels (<50 µm in diameter) with disease relevant lengths and high areal densities (>100 channels per mm^2^) remains an area of active interest. Here, we utilized a patterning technique called magnetic templating that utilizes size-tunable sacrificial magnetic porogens to form aligned microporous structures in of varying diameters inside hydrogels. The role of microstructure size on hydrogel physical properties and the influence of channel size on hydrogel mechanics and Schwann cell behavior was evaluated. Microchannel morphology, assessed by fluorescence microscopy, closely followed the structure of the sacrificial porogen chains, visualized *via* nano computed tomography. Porogens cleared within 7 days and their size had a minor influence on the mechanical properties. Smaller porogens led to higher areal channel densities but resulted in shorter channel lengths. Lastly, *in vitro* experiments suggest Schwann cells organized into tubular structures within smaller diameter channels but displayed mixed morphologies (stretched, clumped, round) in the larger channels. This work demonstrates that magnetic templating enables formulation of hydrogels with aligned microchannels of tunable size with the potential to guide cellular organization.

## Abstract 2

Peripheral nerve injury is a common condition and the development of materials that enhance Schwann cell migration to aid in regeneration is critical as the body has a limited ability to repair transected nerves.

## Graphical abstract

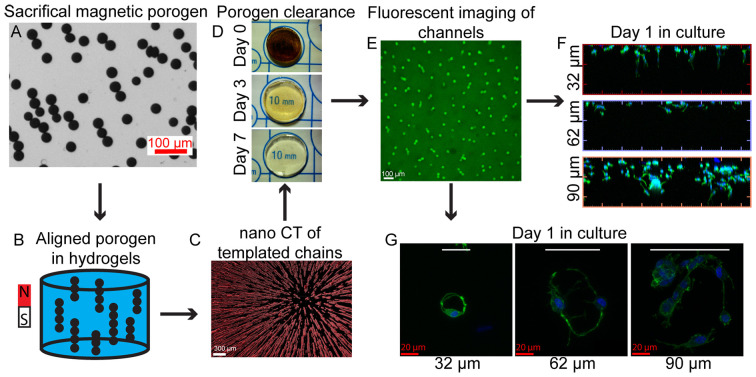

## 1. Introduction

Peripheral nerve injury repair is a common clinical practice. Millions of people are affected by peripheral neuropathy, with an estimated >500 000 peripheral nerve procedures performed annually in the U.S. This condition can lead to impaired sensory, motor or autonomic function depending on the severity of the injury.^1^ Furthermore, nerve injuries increasingly affect young adults and therefore carry lifelong implications for the patient and increased burden on the health care system.^2,3^ Although the peripheral nervous system can spontaneously regenerate after injury, particularly for short defects (less than a few centimeters), this process has complex mechanisms and axons often fail to regenerate fully in large defects. Repairing a large injury requires bridging the defect with a scaffold that serves to guide regenerating cells.^4,5^ This procedure remains clinically challenging, especially if the gap is considerably long (>2 cm). Autologous nerve grafts (autografts) remain the gold standard for peripheral nerve repair due to their superior biocompatibility and retention of donor Schwann cells, which play a crucial role in post-injury repair. However, autografts have several drawbacks, including the need for a second surgery, potential donor site morbidity or neuroma formation, limited availability, and possible size and shape mismatches.^6–8^ Efforts in tissue engineering aim to formulate biomaterial scaffolds that can provide contact guidance and promote nerve reconnection over injury sites.

In peripheral nerve tissue engineering, understanding how Schwann cells behave and respond to biomaterials scaffolds is essential, as these cells play a key role in the repair process by transitioning between myelinating and repair phenotypes.^9^ Upon nerve damage, Schwann cells can play multiple roles in repair. Schwann cells migrate into the nerve gap from sites proximal and distal to the injury and modulate regeneration by promoting axonal survival, clearing axon and myelin debris, secreting signaling molecules that recruit macrophages, and guiding axonal regrowth, followed by re-differentiation to a mature cell prior to remyelinating axons.^9–12^ While the repair SC phenotype is highly mobile, biomaterial chemistry and physical architecture play critical roles in promoting SC motility and can effectively be used to regulate SC behavior and migration.^13–18^

Hydrogels are a popular choice as biomaterials for peripheral nerve repair because of multiple desirable properties that allow them to mimic the extracellular matrix, including their hydrophilicity, porosity, mechanical tunability, biodegradability, and capacity to be chemically modified with biological factors or loaded with agents that enhance cell–material interactions.^19^ Hydrogel-based biomaterial approaches have employed multiple polymers, both synthetic and natural, formulated into hydrogels to promote regeneration upon injury.^20–23^ However, hydrogels are still not as effective as autologous grafts. This is partly because without further processing, hydrogels often possess irregular three-dimensional porosity, which is uncharacteristic of native nerve tissue and does not support proper cell organization and aligned migration.

Various processing strategies have been employed to introduce anisotropic microstructures into biomaterials for peripheral nerve repair, each influencing Schwann cell behavior in different manners. Micromolding and soft lithography techniques have demonstrated that aligned micropatterned topographies direct Schwann cell orientation in 2D, resulting in elongated cell nuclei, and cytoskeleton alignment with the patterned surface.^24–26^ However, this 2D patterning does not recapitulate the complex 3D environment cells encounter in native tissue. Electrospun nanofiber scaffolds have been shown to promote both Schwann cell alignment and proliferation through an aligned fiber network.^27,28^ However, electrospun nanofibrous materials are 2D and typically utilized for developing conduits that are hollow, therefore the conduit would require a second filler material. Additionally, the electrospinning technique can be challenging to apply for some polymer chemistries due to issues with solubility or viscosity. In some cases, potentially cytotoxic solvents are needed to expand the range of applicable biomaterials.^29–31^

While prior research has explored Schwann cell morphology on 2D substrates, there has been limited investigation into their behavior in 3D scaffolds with aligned microarchitecture. Studies using techniques like wire templating,^32^ fiber templating,^33^ uniaxial freeze drying,^34^ alginate capillary self-assembly within molds,^35,36^ and 3D printing,^37,38^ aim to create parallel channels or pores within 3D scaffolds. Reports of these techniques have shown that microchannel alignment and porosity may impact Schwann cell behavior and that Schwann cell migration can be influenced by the diameter of these channels, with larger channel diameters commonly leading to higher numbers of infiltrating cells. However, these techniques result in either low channel density or length, and characterization of such physical architecture has been limited, hindering correlations of how microstructure may influence cell behavior. Further, studies of Schwann cell migration depth or morphological changes in response to architecture diameter or length remain limited. For example, work accomplished using wire or fiber templating has led to hydrogels with large channel length spanning 1–2 cm, but low areal densities of channels (<4 channels per mm^2^) and such properties are not always explicitly reported.^32,33,39,40^ Furthermore, wire and fiber templating techniques commonly lead to pore diameters greater than 100 µm, which is much larger than the pores occupied by peripheral nerves or the channels remaining in decellularized nerve allografts.^41–43^ Uniaxial and unidirectional freeze-drying methods have yielded smaller microchannel diameters, as low as 20 µm, at higher areal densities (∼72 channels per mm^2^). However, channel length characterization is seldom reported, despite being a crucial factor for cell infiltration. Further, unidirectional freeze-drying can result in inconsistent pore structure and pore size variation across the hydrogel.^44–46^ Alginate capillary self-assembly has enabled very high areal density of channels, reaching up to 600 channels per mm^2^ with pore sizes as small as 20 µm;^47^ however, the length of these pores is often not reported and the use of a diffusion gradient to form the capillaries imposes limits on their length and on the speed with which long microchannels can be formed. Lithography techniques have been used to generate microchannels in PDMS and these have been rolled into cylindrical nerve repair conduits with channel cross sectional areas spanning 50 µm × 100 µm to 150 µm × 100 µm and pore areal densities of 44 to 80 channels per mm^2^, with observed effects in axon migration 8-weeks after implantation in a rat transected sciatic nerve model.^48^ Lastly, 3D printing techniques have been used to prepare micropatterns, but they are typically involved as part of other techniques for more complex scaffold design. For example, 3D printed materials have been uniaxially frozen,^49^ or have been used as filler materials in electrospun conduits,^50^ resulting in patterned materials with varying porosities. For example, 3D printing produced randomly distributed small pores in scaffolds ranging in size from 15 to 35 µm.^51^ In other instances, 3D printing has been used to obtain hydrogels with aligned pores ranging from 150–1500 µm in diameter and spanning 1–2 cm, albeit with low channel numbers (∼fewer than 10 channels in the entire structure) and unreported areal channel density.^37,38,49,52^Table 1 offers a summary of important morphological characteristics of channels or pores in biomaterials intended for peripheral nerve regeneration, suggesting that current methods suffer from tradeoffs between channel density, length, and diameter. Therefore, techniques that allow for millimeter to centimeter length channels with high areal density of cell-scale pores (>1000 channels per mm^2^) remain of interest. Further, investigating the relationship between these physical characteristics and SC behavior in well characterized 3D microenvironments is needed as these physical characteristics shift the balance between cellular migration and their phenotypic organization.

**Table 1:** Summary of peripheral nerve biomaterials with 3D micropatterning

Method	Pore diameter (µm)	Pore length (mm)	Pore areal density (# mm^−2^)	Ref.
Wire templating	450, 600	2	NR	32
Fiber templating	200	15	NR	33
	110, 500		NR	39
	100, 200	20	NR	40
	105	NR	NR	53
Unidirectional freezing	125	NR	72	34
	20–80	NR	NR	54
	10–300	NR	NR	55
Alginate capillary self-assembly	27	NR	500	36
	10–77	NR	60–630	35
Lithography/PDMS	50 µm × 100 µm to 150 µm × 100 µm	3 mm	44–80	48
3D printing	400–1500	10	NR	37
	150	NR	6	38
	50, 500	19	NR	49
	1200–2000	5	NR	52
	4–8	85–155	NR	56
Magnetic templating	0.1–20	>1^b^	NR	57
	68^a^	>1^b^	NR	58
	65^a^	NR	NR	59

a Values reported were not of the actual measured pore diameter but of the porogen size.

b Values reported are not of the actual measured pore length, but of the mold size used.

Prior work from our group obtained hydrogels with highly aligned microchannel structures using a micropatterning technique called magnetic templating, in which sacrificial magnetic alginate microparticles (MAM) are aligned into chains and then removed to leave behind oriented channels within a 3D hydrogel matrix.^57–59^ Schwann cell migration was evaluated *in vitro* in non-templated hydrogels and in hydrogels templated with MAMs of ∼60 µm diameter in the presence of a growth factor gradient.^58^ Schwann cells penetrated the templated hydrogels but not the non-templated hydrogels and the growth factor gradient resulted in increased migration depth. Further, in other work, magnetically templated hydrogels guided axon extension from dorsal root ganglia *in vitro*.^57^ Prior work has also studied MAM alignment quality as a function of the rheological properties of the precursor hydrogel solution in granular hydrogel composites.^60^ While magnetic templating has shown potential in guiding cell migration, the influence of porogen size, and subsequently pore size on Schwann cell migration and morphology has not been studied and is a key factor in determining the potential of this approach and of the resulting biomaterials for peripheral nerve repair.

In this work, we investigated the role of MAM porogen diameter on the microarchitecture of magnetically templated hydrogels and evaluated the role of this microarchitecture on Schwann cell morphology in the channels. To do this, we formulated three different sized MAMs and used them to prepare magnetically templated hydrogels of hyaluronic acid and collagen. These hydrogels were comprehensively characterized in terms of channel diameter, length, areal and volumetric density, and channel interconnectivity pre- and post-porogen clearance. Porogen clearance was quantitatively assessed using magnetic particle imaging, a non-invasive approach to quantify the mass of magnetic nanoparticles in a sample. Mechanical properties were characterized *via* indentation. Lastly, Schwann cell migration and resulting morphological phenotypes were evaluated.

## 2. Materials and methods

### 2.1. Materials

Iron oxide nanoparticles of ∼30 nm in hydrodynamic diameter and coated in poly (ethylene glycol) were acquired from Ferrotec (Livermore, California, USA). Novec 7500 fluorocarbon oil (N17501) was purchased from Gallade Chemicals. Low-viscosity alginate (A1112), ethylenediamine tetraacetic acid powder (EDS-100G), Fluorinert™ FC-70 fluorocarbon oil (F9880), 1.5–1.8 MDa hyaluronic acid sodium salt (53747), triethylamine (T0886), glycidyl methacrylate (770342), lithium benzyl pentanoate photoinitiator (900889), forskolin (F3914), and fluorescein isothiocyanate–dextran (52471) were purchased from Sigma-Aldrich. Acetone (A18-20), calcium chloride (C79-500), and 0.1 M EDTA solution (SS412-1) were purchased from Fisher Scientific. Collagen I (354249) and DMEM (MT10013V1) purchased from Corning. Rat Schwann cells were procured from Sciencell (R1700). Fetal bovine serum (10438026), bovine pituitary extract (13028014), and fibroblast growth factor (PHG0264) were acquired from Gibco. Penicillin–streptomycin–amphotericin B was obtained from MP Biomedicals (091674049). All chemicals and materials were used as received according to manufacturer's instructions.

### 2.2. Equipment and software

The flow-focusing microfluidic chip (3200515) and pressure controlled Mitos P-pumps (3200016) used for droplet generation were obtained from Dolomite microfluidics. Particles were imaged in a Keyence BZ-X710 microscope (Keyence Corporation, Itasca, IL, USA). An XRadia 620 Versa computed tomography scanner was used to image chain alignment (Carl Zeiss X-Ray Microscopy, Oberkochen, Baden-Württemberg, Germany). VGStudio Max (Volume Graphics GmbH, Heidelberg, Germany) and IMARIS (Oxford Instruments) were used for post-processing and quantification of nano computed tomography (nano CT) images. Magnetic particle imaging (MPI) in a MOMENTUM™ imaging scanner (Magnetic Insight, Alameda, CA, USA) was conducted using a custom-made MPI bed and the standard user interface with high sensitivity mode (gradient field: 3.0 T m^−1^; excitation field 45 kHz, 20.5 mT *z*-amplitude, 15.5 mT *x*-amplitude; field of view: *z* = 12 cm, *x* = 6 cm) available in the default software of the MOMENTUM™ imager. Mechanical characterization was performed using a Bruker BioSoft *In situ* Indenter (Bruker, Billerica, MA, USA). A Zeiss LSM 880 laser-scanning confocal microscope was used for imaging (Carl Zeiss Confocal Microscopy, Oberkochen, Baden-Württemberg, Germany). An asymmetrical mixer was used for material mixing (Flacktec, DAC 150.1 FVZ). Hydrogel cylindrical molds were obtained from Grace Biolabs (6642011).

### 2.3. Magnetic alginate microparticle (MAM) fabrication and magnetization measurements

Magnetic alginate microparticles were fabricated as previously described.^61^ Briefly, a droplet phase composed of alginate, calcium ethylenediamine tetraacetic acid (CaEDTA), and iron oxide nanoparticles at 75 mg mL^−1^ were introduced into a flow focusing microfluidic chip, where it encountered a pinching solution of either Novec 7500 fluorocarbon oil or Fluorinert FC-70 fluorocarbon oil. The flow rates of both phases were adjusted to obtain the desired droplet size. Droplets were collected in a crosslinking solution composed of acidified oil under stirring. Finally, particles were washed with acetone and placed in a secondary crosslink solution containing calcium chloride for 1 min before rinsing with and storing in water at 4 °C until use. Aggregates were removed by sedimentation and pipetting the supernatant. This process was repeated multiple times until obtaining suspensions of microparticles with no visible aggregates. The microparticles in the supernatant were lyophilized, and the dried mass was measured to estimate MAM loss. Finally, MAM solution volumes were adjusted to create an 11% v/v stock.

### 2.4. Glycidyl methacrylate hyaluronic acid (GMHA) synthesis

GMHA was synthesized as previously described.^62^ Briefly, hyaluronic acid sodium salt was dissolved in 50% v/v acetone in water at 10 mg mL^−1^ at room temperature. The dissolved solution was incubated with 6.7% triethylamine for 20 min, and subsequently reacted with 6.3% glycidyl methacrylate for 24 h at room temperature. The product was precipitated in 20× volumetric excess of acetone and redissolved in 50 mL of water. The precipitation and redissolution process was repeated once more. Methacrylated hyaluronic acid underwent dialysis against 1× phosphate-buffered saline (PBS) for 48 h, followed by dialysis against water for 24 h using a 10 kDa molecular weight cutoff dialysis cassette. The GMHA product was sterile filtered, lyophilized for 7 d, and stored at −20 °C with desiccant until use. This protocol yields methacrylation degree of 16%.

### 2.5. Magnetically templated hydrogel fabrication for mechanical and *in vitro* assessment

Magnetically templated scaffolds were prepared as previously described.^59,63,64^ Hydrogels were fabricated with 10 mg ml^−1^ GMHA and 3 mg mL^−1^ collagen I with channels parallel or perpendicular to hydrogel surface for mechanical testing of anisotropic properties. GMHA was dissolved in 0.3% v/v lithium phenyl-2,4,6-trimethylbenzoylphosphinate photo initiator solution overnight at room temperature. Hydrogel solutions were subsequently completed with collagen I, mixed in an asymmetrical mixer at 3500 rpm for 5 s and then 11% v/v MAM stock was added for a final concentration of 2.2% v/v MAMs and mixed again with a positive displacement pipette. Three groups were studied representing hydrogels templated with 32, 62, and 90 µm MAMs respectively.

The complete hydrogel solution was loaded into a positive displacement pipette and injected into 8 mm diameter × 1.7 mm height cylindrical molds. The molded hydrogel solutions were placed within a 90 mT magnetic array at 4 °C for 30 min to allow for MAM chain alignment. Hydrogels templated with unaligned MAMs were formulated as a control by crosslinking hydrogels that skipped the magnetic field application step thereby leading to homogeneous distribution of MAMs that possess no interconnectivity; hence, no channels upon their clearance. Then, hydrogels were placed under a 365 nm UV light with 18–20 mW cm^−2^ intensity for 10 min to photocrosslink the GMHA, followed by incubation at 37 °C for 35 min to allow collagen fibrillogenesis. After hydrogel fabrication, MAM clearance was conducted by placing hydrogels in 0.1 M EDTA solution for 7 d at 37 °C on a shaker (70 rpm). EDTA solutions were changed daily until complete MAM dissolution and cleared hydrogels were then stored in 1× PBS until use.

### 2.6. Characterization of chain formation on templated hydrogels through nano computed tomography (nano CT)

Nano computed tomography was used to assess the formation of MAM chains in magnetically templated hydrogels prior to MAM clearance. An XRadia 620 Versa tomography scanner was used. Scans were conducted at a voltage of 70 kV, a current of 121 µA for a power of 8.5 W. Voxel sizes for *x*, *y*, and *z* dimensions was 7–9 µm. Post-processing of reconstructed scans was conducted using VGStudio Max™. Reconstructed scans were then imported to IMARIS as TIFF stacks for quantification of chain diameter, length, density, and connectivity.

### 2.7. Quantitative assessment of MAM clearance using magnetic particle imaging (MPI)

Magnetically templated hydrogels in triplicate were placed into separate 35 mm diameter Petri dishes. Each Petri dish was then placed in a custom made MPI bed such that the Petri dish was centered in the MPI field of view for image acquisition. MPI images were acquired in a MOMENTUM™ imager using the ‘High Sensitivity’ isotropic mode, having a 3.0 T m^−1^ gradient strength and excitation amplitudes in the *X* and *Z* directions of 15.5 mT and 20.5 mT, respectively. The gels were then placed into 0.1 M EDTA solution and heated/rotated as described earlier in section 2.5. At specific timepoints, namely 1, 6, 24, 48, 72, 120, and 168 hours after EDTA exposure, the gels were removed from the EDTA solution, placed into a Petri dish, and imaged as described earlier, then placed back into EDTA solution, with EDTA changes occurring every 24 hours.

The sharpened MPI scans, in DICOM format, were loaded into 3D Slicer, an open-source imaging computing platform (slicer.org).^65^ The maximum signal of each image was determined, then a boundary at 50% of this maximum signal defined a region of interest (RoI). The pixel values within this RoI were averaged then multiplied by the number of pixels in the RoI and reported as the total signal. The total signal value of each MPI image was normalized by that of the initial MPI scan for that specific hydrogel. The values were then grouped by MAM size (32, 62, 90 µm) and plotted as average normalized total signal *versus* time, with error bars representing standard deviation (*n* = 3).

### 2.8. Mechanical characterization of magnetically templated hydrogels *via* indentation

Stress–relaxation measurements of templated and non-templated hydrogels were obtained *via* non-destructive bulk indentation testing using a Bruker BioSoft *In situ* Indenter. Tests were performed by indenting 7.5% (150 µm) of the total sample height at a rate of 20 µm s^−1^. The probe was held at the maximum indent depth to obtain stress relaxation data; hold times were kept at 40 s to allow samples to reach a quasi-static state. All measurements were performed with a 3 mm-diameter spherical glass tip. Samples were kept hydrated and analyzed without submersion. Three locations were analyzed per sample, with each experimental group containing six samples (*n* = 6). As described by Stewart *et al.*, the relaxation data were converted into a time-dependent relaxation modulus from force–displacement data using the Hertz contact model accounting for a parabolic contact region.^66^ The standard linear solid model for viscoelasticity was used to fit relaxation data to obtain the rate-dependent instantaneous modulus and the steady-state relaxation modulus.

### 2.9. Microchannel visualization after MAM clearance and determination of channel physical characteristics

Magnetically templated hydrogels were fabricated as described in section 2.3 and channels were later backfilled by placing gels in 1 mg mL^−1^ of 2 MDa fluorescein isothiocyanate-dextran and incubating for 3 h at 37 °C. Solutions were then removed and hydrogels washed with 1× PBS every 30 min for 2 h. Images of magnetically templated hydrogels were acquired at 5× magnification on a Zeiss LSM 880 laser-scanning confocal microscope with ∼1200–1500 µm *z*-stack at a 15 µm step size. Channel length distributions, channel volume density, and average channel diameter were quantified as a function of MAM size using IMARIS.

### 2.10. *In vitro* evaluation of rat Schwann cells cultured in templated hydrogels

For *in vitro* culture, hydrogels underwent a sterile 1× PBS equilibration for 1 d, followed by equilibration in complete Schwann cell media for 1 d (10% fetal bovine serum, 1% penicillin–streptomycin–amphotericin B [MP Biomedicals, 091674049], 20 µg mL^−1^ bovine pituitary extract, 4 µM forskolin, and 10 ng mL^−1^ fibroblast growth factor in Dulbecco's Modified Eagle's medium).

Passage 2 rat Schwann cells were seeded on hydrogel samples at a seeding density of 20 × 10^3^ cells per cm^2^ of hydrogel surface in complete rat Schwann cell media. Cell media was changed every two days, and the rat Schwann cells were cultured for one or three days. At the end point, cells were fixed in 4% paraformaldehyde for 1 h and washed with 1× PBS every hour for 3 h. Fixed samples were co-stained with Hoechst 34580 (1 : 5000 dilution) for nuclei and Alexa Fluor 488 Phalloidin (1 : 1600 dilution) for actin filament in 1× PBS for 30 min. After staining, hydrogel samples were washed with 1× PBS for 30 min, three times and then stored in 1× PBS at 4 °C until imaging. Fixed and stained hydrogel samples were placed in a 35 mm diameter glass-bottom dish and imaged using 10× and 25× magnification on a Zeiss LSM 880 laser-scanning confocal microscope. The resulting czi files were imported into IMARIS for analysis of cell count, migration, and morphology.

### 2.11. Image analysis

IMARIS or ImageJ was utilized to analyze multiple data sets by importing tiff stacks or czi files of the acquired images.^67^ For analysis of nano CT data, tiff stacks of the chains were imported, converted into an IMARIS native file and regions of interest were segmented using the cropping function. Surfaces were created around chains based on contrast differences between the object and the background. Subsequently, a masked intensity channel was created from the generated surfaces, and this masked channel was used to form filaments using the filament tracing module in IMARIS. Chain statistics (*i.e.* length, diameter, branch points) were obtained from the traced filament results. For areal counts of chains, Image J was utilized to process tiff stacks. Images were binarized, threshold for circular objects and chains counted as a function of depth.

For analysis of confocal data corresponding to backfilled templated hydrogels, czi files and tiff stacks were imported and converted into an IMARIS native file. For microchannel statistics, surfaces were created around chains based on contrast differences between the object and the background. Subsequently, a masked intensity channel was created from the generated surfaces and this masked channel was used to form filaments using the filament tracing module in IMARIS. Chain statistics (*i.e.* length, diameter, branch points) were obtained from the traced filament results. To determine channel areal density, Image J was utilized to process tiff stacks. Images were binarized, thresholded for circular objects, and chains counted as a function of depth.

For analysis of confocal data corresponding to Schwann cell migration into templated hydrogels, czi files were imported into IMARIS and converted into IMARIS native file. Spot objects were created to identify cell nuclei and cell count as a function of depth were obtained from this data. Surfaces were created around the cell nuclei and the cell actin as two separate objects to distinguish cells in the higher magnification data.

### 2.12. Statistical analysis

MAM size distributions were obtained by analyzing optical images taken with a 4× objective in a Keyence BZ-X710 microscope. Images were processed using MATLAB R2021a, in which images were binarized using Otsu's threshold method for image segmentation and an optimal grayscale threshold is identified between background and foreground. After binarization, thresholding was used on foreground objects to retain circular objects with a diameter above 1 µm and to retain objects with high shape uniformity using an elliptic Fourier coefficient above 5. After segmentation of the population of particles, areas were obtained from the pixel-to-µm calibration from the image, and a corresponding diameter was determined. Diameters were then fitted to a log–normal size distribution. Statistical differences on mechanical indentation data were obtained by performing a 2-way ANOVA and contributions of the independent parameters to statistical differences were acquired from Tukey's *post-hoc* analysis. One way Brown-Forsythe ANOVA was used for comparing statistical differences between the templated hydrogels and the tissue control. Statistical differences in volume density of channels were obtained by performing a 1-way Welch's ANOVA.

## 3. Results

### 3.1. Microfluidic formulation yields MAM porogens with tunable size, narrow size distribution, and consistent magnetic properties

**Fig. 1:**
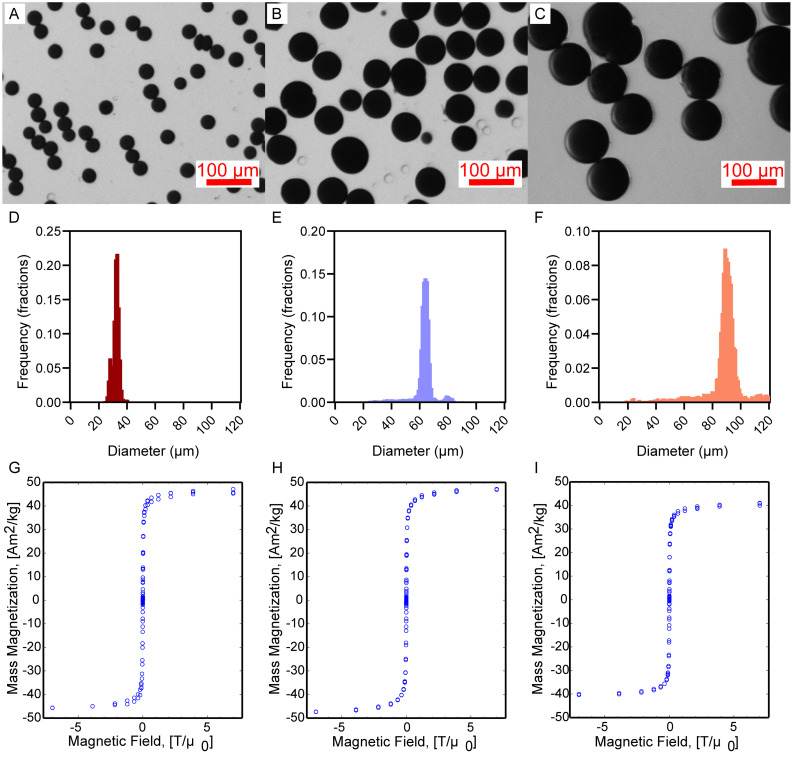
Microfluidic formulation yields MAMs with tunable sizes, narrow size distribution, and consistent magnetic properties. Keyence optical images of (A) 32 µm, (B) 62 µm, and (C) 90 µm MAMs. Lognormal fitted size distributions of (D) 32 µm, (E) 62 µm, and (F) 90 µm MAMs. The number of particles used to obtain size distributions were 60 585, 9125, 5414, respectively. Magnetization curves for (G) 32 µm, (H) 62 µm, and (H) 90 µm MAMs in solid samples.

Fig. 1A–C shows optical images of the formulated MAMs with three distinct sizes. The homogeneous contrast arising from the encapsulated iron oxide suggests that the nanoparticles are homogeneously distributed and stable within the MAMs.^61^ Clear bubbles in Fig. 1B are attributed to some remaining oil droplets. The MAMs have narrow size distributions, as shown in Fig. 1D–F, with average diameters of 32 µm, 62 µm, and 90 µm and corresponding coefficients of variance below 5% (Table 2) for all groups. The MAMs are also highly spherical and the fraction of non-spherical particles throughout the groups is small and would not interfere with alignment. Magnetometry showed a saturation specific magnetization of 36–41 Am^2^ kg^−1^ across all groups. These values show consistent encapsulation of iron using microfluidics, albeit with some loss of the initially loaded iron. Since the saturation specific magnetization of free particles was 84 Am^2^ kg_Fe_3_O_4__^−1^ (or 117 Am^2^ kg_Fe_^−1^) (Fig. S1, SI) it would mean MAMs are ∼43–49% Fe_3_O_4_ by mass. This value represents a decrease from the expected 88% based on the loaded iron and alginate masses in the droplet phase.

**Table 2:** MAM sizing and saturation specific magnetization

Group	Oil : droplet flow rates (µL min^−1^)	Average MAM diameter (µm)	Standard deviation (µm)	Coefficient of variance (%)	Saturation specific magnetization (*M*_s_) (Am^2^ kg^−1^)
32 µm	75 : 7	31.7	1.5	4.7	41.0
62 µm	30 : 10	62.3	2.3	3.7	41.8
90 µm	15 : 10	90.2	4.0	4.4	36.2

### 3.2. MAM diameter exerts a strong influence on the physical characteristics of chains formed under applied magnetic fields in pre-hydrogel solutions

**Fig. 2:**
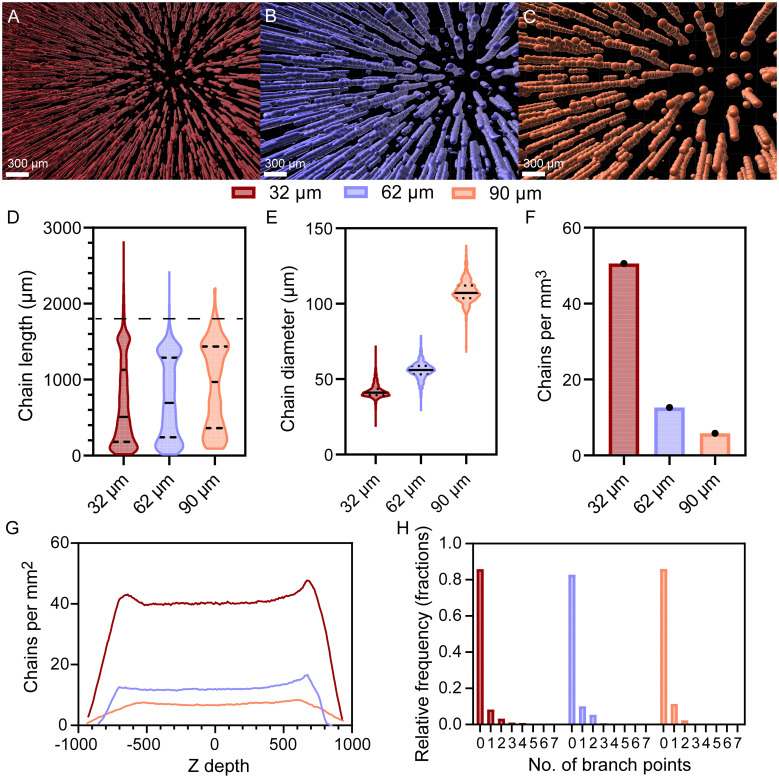
MAM diameter exerts a strong influence on chain length, diameter, areal density, while retaining uniform areal density and low degree of branching. Representative nano CT reconstruction in IMARIS of (A) 32 µm, (B) 62 µm, and (C) 90 µm MAM templated hydrogels. (D) Filament length distribution of templated gels. Dashed line represents the hydrogel thickness. (E) Chain diameter distributions. (F) Volume density of chains. (G) Areal density of chains. (H) Distributions of number of branches per chain. The number of chains with branch points above 5 where below 1%. One whole hydrogel per group was analyzed with nano CT. Solid lines represent the mean and dotted lines represent lower and upper quartiles in violin plots.

Magnetically templated hydrogels were kept inside molds and imaged *via* nano CT to visualize chain alignment in a 3D volume. Nano CT image stacks were analyzed in IMARIS to evaluate MAM chain physical characteristics (Fig. 2). Reconstructed nano CT images show an increase in the number of chains as the MAM size used in templating decreased (Fig. 2A–C). Chain length distributions were quantified and IMARIS analysis showed an increase in the median chain length, with values of 507, 692, and 970 µm for gels templated with 32, 62, and 90 µm MAMs, respectively (Fig. 2D). Furthermore, 19, 20, and 33% of the chains span 75% of the hydrogel thickness for gels templated with 32, 62, and 90 µm, respectively. Chain diameters were estimated based on the length and volume, assuming a cylindrical shape, and the resulting values closely matched the corresponding MAM diameters, with chain diameters of 42, 56, and 108 µm for gels templated with 32, 62, and 90 µm MAMs, respectively (Fig. 2E). Volume density (Fig. 2F) and areal density (Fig. 2G) of chains also vary with MAM size. The volume density of chains was determined to be 50, 12, and 5 chains per mm^3^ and areal densities were determined at 41, 12, and 7 chains per mm^2^ for hydrogels templated with 32, 62, and 90 µm MAMs, respectively. The areal density plot in Fig. 2G shows a sharp decrease in areal density at both edges of the gel due to slight angle differences in the stacks that led to some edge chains being off the plane.

For each group, a few chain lengths were observed above 1800 µm, which corresponds to the full thickness of the gel. We attribute this to some chains having branches and IMARIS calculating length as the summed lengths of the main chain and its branches. Thus, some branched chains led to summed lengths larger than the hydrogel thickness. However, this corresponds to a small fraction of the total number of chains, with only 3%, 1% and 2% of the chains being longer than 1800 µm for gels templated with 32, 62, and 90 µm MAM, respectively. Additionally, calculations of the distribution of the number of branch points per chain show that 85%, 83%, and 85% of the chains have no branch points in gels templated with 32, 62, and 90 µm MAMs (Fig. 2H). Thus, most chains form highly aligned single columns.

The total volume fraction occupied by chains with respect to the total hydrogel volume was also estimated by adding the individual volume of each chain and dividing by the total hydrogel volume. The porous volume created by the chains was determined to be 5.0, 2.0, and 4.9% v/v for hydrogel templated with a 32, 62, and 90 µm MAM, respectively.

### 3.3. Magnetic alginate microparticle porogen diameter does not influence clearance from magnetically templated hydrogels

**Fig. 3:**
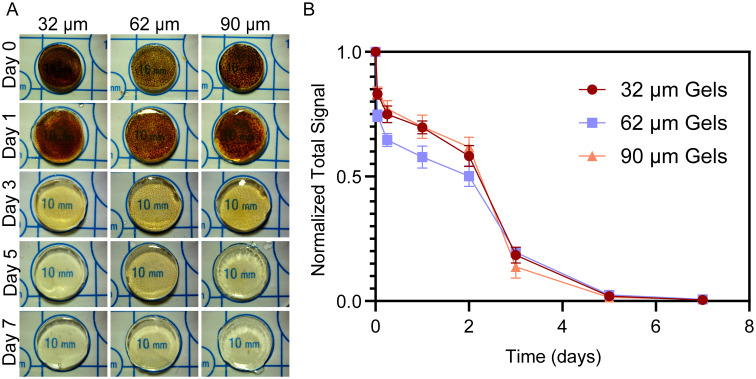
MAM diameter does not affect clearance of magnetically templated hydrogels. (A) Representative daily optical images of MAM clearance from all templated hydrogel groups. (B) Remaining MPI normalized average total signal per day for each MAM group (*n* = 3).

MAM clearance from magnetically templated hydrogels was carried out and assessed both qualitatively and quantitatively using optical microscopy and magnetic particle imaging (MPI). The templated hydrogels were placed in 0.1 M EDTA solutions to chelate the calcium ions that crosslink the alginate and images of each hydrogel were taken every 24 h before replacing the EDTA solution. Optical microscopy images show clearance of the MAMs by day 7 regardless of the MAM size used during the templating process (Fig. 3A). A quantitative assessment of MAM clearance was performed using MPI (Fig. 3B) to track signal loss due to iron mass removal from the gels. MPI measurements show that by day 7, the signal is less than 1% of the initial signal. The signal was not quantifiable after 7 days. Quantitative assessment suggests two stages of burst release for the MAMs. Importantly, qualitative and quantitative assessments suggest that MAM diameter does not affect clearance rate from magnetically templated hydrogels.

### 3.4. Porogen diameter exerts a strong influence on the physical characteristics of channels formed after MAM clearance

**Fig. 4:**
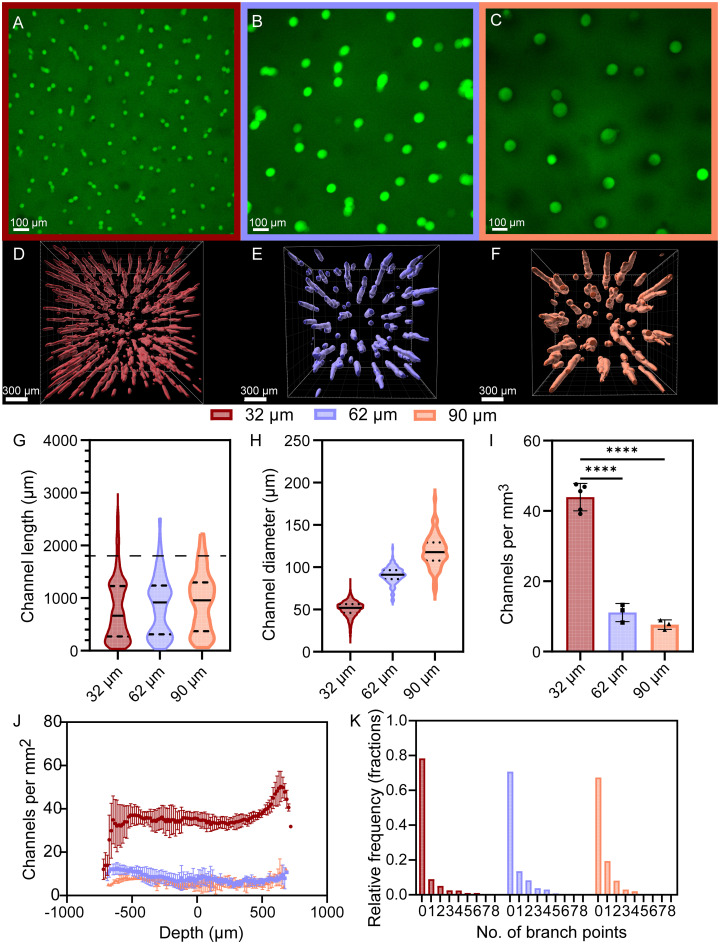
Characterization of microchannels after porogen clearance. Confocal fluorescence visualization of the areal density of microchannels in FITC-dextran backfilled hydrogels templated with (A) 32, (B) 62, and (C) 90 µm MAMs. Top view of microchannel surfaces obtained with IMARIS for channels templated with (D) 32, (E) 62, and (F) 90 µm MAMs. Distributions of (G) microchannel lengths, (H) channel diameter, (I) microchannel volume density, (J) microchannel areal density, and (K) number of branch points per channel. The number of channels with branch points above 5 where below 1%. One-way ANOVA followed by a Tukey's *post-hoc* analysis was carried out. *P*-values: * < 0.05, ** < 0.005, *** < 0.0005, **** < 0.00005.

To visualize microchannel architecture, templated hydrogels were backfilled with FITC-dextran (2 MDa) after MAM dissolution and *z*-stacks were obtained using confocal microscopy at 15 µm intervals. Qualitative observations of 2D images from templated hydrogels show differences in the number of channels (Fig. 4A–C). *Z*-stacks were imported into IMARIS, and surfaces were created based on the fluorescence intensity (Fig. 4D–F). As with nano CT data, channel length was characterized for each group (*n* = 3) and results show a median channel length of 663, 889, and 957 µm for hydrogels templated using MAMs with diameters of 32, 62, and 90 µm, respectively (Fig. 4G). For some channels, IMARIS calculated a length above 1800 µm, which is the full thickness of the gel. As with the nano CT image analysis we attribute this to some channels having branches. These channels still represent a small fraction, with 6%, 5%, and 8% of the total number of channels being longer than 1800 µm for gels templated with 32, 62, and 90 µm MAMs, respectively. Statistical analysis of the individual replicates (Fig. S2) shows there were no statistically significant differences in the distributions of channel length within the gels of each group, suggesting the magnetic templating process yields hydrogels with reproducible chain length distributions for the MAM diameters used in these studies. Channel diameters were estimated from their determined length and volume, assuming a cylindrical shape, and were observed to increase with increasing MAM diameter, resulting in channels with diameters of 51, 90, and 118 µm for gels templated with 32, 62, and 90 µm diameter MAMs, respectively. This corresponds to an increase of 27–45% in diameter compared to MAM diameter (Fig. 4H). Compared to diameters obtained by nano CT for MAM chains, the diameters of the backfilled channels are larger, which is attributed to potential diffusion of the fluorophore at the walls of the channels towards the bulk of the hydrogel. Volume density (Fig. 4I) and areal density (Fig. 4J) of channels vary with MAM size. The average volume density was determined to be 44, 11, and 8 channels per mm^3^ for hydrogels templated with 32, 62, and 90 µm MAMs, respectively. Statistically significant differences in volume density were observed between hydrogels templated with 32 µm MAMs relative to the other groups. No statistically significant difference was observed between the 62 and 90 µm MAM templated groups. Areal densities were determined to be 35, 8, and 6 channels per mm^2^. Analysis of the distribution of branch points suggest that 78%, 70%, and 67% of the channels have no branch points in gels templated using 32, 62, and 90 µm diameter MAMs (Fig. 4K). Table 3 compares median length, volume density, and areal density obtained for MAM chains and hydrogel channels using nano CT and confocal microscopy, respectively.

**Table 3:** Summary of the determined physical characteristics of micropatterned hydrogels as assessed *via* nano CT and fluorescent microscopy

Group	Median chain length from nano CT (µm)	Median channel length from fluorescence (µm)	Volume density from nano CT (chains per mm^3^)	Volume density from fluorescence (channels per mm^3^)	Areal density from nano CT (chains per mm^2^)	Areal density from fluorescence (channels per mm^2^)
32 µm	507	663	50	44	41	35
62 µm	692	889	12	11	12	8
90 µm	970	958	5	8	7	6

The volume fraction occupied by the channels with respect to the total hydrogel volume was also estimated by adding the individual volume of each channel and dividing this by the total hydrogel volume. The porous volume created by the channels was determined to be 7.9, 5.9, and 7.8% v/v for hydrogels templated with a 32, 62, and 90 µm MAM, respectively. These volume fractions show an increase with respect to the volume fractions estimated by nano CT, however, they scale with the already noted increase in channel diameter compared to chain diameters determined.

### 3.5. Magnetic alginate microparticle diameter does not exert a strong influence on the anisotropic mechanical properties of the magnetically templated hydrogels

**Fig. 5:**
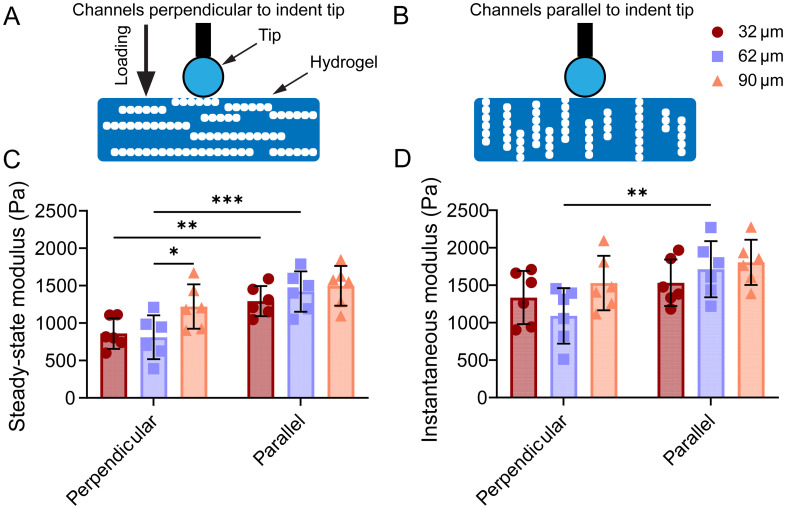
MAM diameter does not exert a strong influence on the anisotropic mechanical properties of magnetically templated hydrogels after porogen clearance. Representative diagrams of indentation on a (A) perpendicular and (B) parallel templated hydrogel. (C) Comparison of steady-state modulus (*n* = 6) of hydrogels templated with channels perpendicular and parallel to the indent tip. (D) Comparison of instantaneous modulus (*n* = 6) of hydrogels templated with channels perpendicular and parallel to the indent tip. Measurements were analyzed with a 2-way ANOVA for statistical significance and *post-hoc* analysis (Tukey's) for parameter contribution to statistical difference. *P*-values: * < 0.05, ** < 0.005, *** < 0.0005, **** < 0.00005.

Upon MAM clearance, templated hydrogels were evaluated for their mechanical stiffness through quasi-static indentation measurements (Fig. 5A and B). Both the steady-state modulus, which indicates the stiffness of the material after stress relaxation has occurred (long-term, static resistance to deformation), and the instantaneous modulus, which represents the materials response to immediate loading (short-term, dynamic impact), have been characterized to determine potential anisotropy in said properties and how hydrogels compare to native nerve. A statistically significant difference in steady state modulus with templating direction was observed for the hydrogels templated using 32 and 62 µm MAMs (*n* = 6) and alignment direction was determined to be the main contributor to differences in mechanical properties based on 2-way ANOVA and *post-hoc* analysis (Tukey's) (Fig. 5C). The instantaneous modulus only showed a significant difference in the mechanical properties with alignment direction for the hydrogels templated using 62 µm MAMs (Fig. 5D). When compared to the steady-state modulus values of freshly explanted nerve (1.31 ± 0.33 kPa) reported by Lacko *et al.* (Fig. S3),^57^ only the 62 µm templated hydrogel in perpendicular alignment showed a significant difference, with values of 0.81 ± 0.29 kPa. In contrast, the hydrogels templated using the 32 and 90 µm diameter MAMs showed no significant differences from the freshly explanted nerve, with values of 0.86 ± 0.21 kPa and 1.22 ± 0.30 kPa, respectively. Overall, the mechanical characterization of templated hydrogels shows anisotropic properties resulting from the templating process. However, channel size has a limited influence on the mechanical properties with alignment direction being the primary factor contributing to changes in these properties and their anisotropy.

### 3.6. Channel diameter in magnetically templated hydrogels influences Schwann cell migration and morphology

**Fig. 6:**
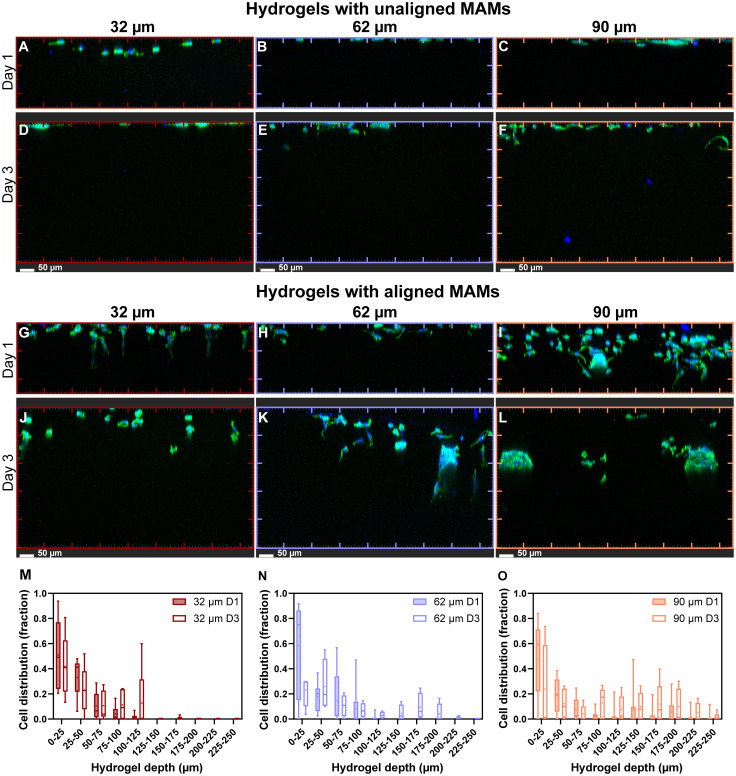
Rat Schwann cell migration into hydrogels with magnetically templated channels is influenced by channel diameter. Migrating rat Schwann cells seeded in hydrogels templated with non-aligned (A, D) 32 µm diameter MAMs, (B, E) 62 µm diameter MAMs, and (C, F) 90 µm MAMs and imaged at (A–C) day 1 and (D–F) day 3 after seeding. Migrating rat Schwann cells seeded in hydrogels templated with aligned (G, L) 32 µm diameter MAMs, (H, K) 62 µm diameter MAMs, and (I, L) 90 µm diameter MAMs and imaged at (G–I) day 1 and (J–L) day 3 after seeding. Cellular distributions in templated hydrogels at day 1 and day 3 for hydrogel templated with (M) 32, (N) 62, and (O) 90 µm MAMs. Cell count data are plotted as the minimum observation, lower 25% quartile (Q1), median, mean, upper 75% quartile (Q3), and maximum observation (*n* = 3 per group). All ticks represent 100 µm intervals.

Rat Schwann cells were seeded at the top of templated hydrogels with channels parallel to the indentation tip and cell migration was evaluated at day(s) 1 and 3 imaged as shown in Fig. S4. Cells were cultured for up to 3 days to evaluate early migration dynamics of Schwann cells into hydrogels. The short time window was chosen as it has been suitable to observe changes in migration and organization of Schwann cells with changes observed daily after seeding,^68^ and peak thrust migration observed in days 2–3.^69^ Confocal microscopy *z*-stacks of hydrogels were acquired at 10× magnification from top to bottom, shown as maximum intensity projections along the depth of the gels for visualization of cellular migration through the depth of the hydrogels (Fig. 6). Maximum intensity projections of 250 µm *z*-stacks projected on their side were taken for day 1 and 500 µm *z*-stacks for day 3, corresponding to the depths at which cells were observed (no cells were observed below these depths for each corresponding day).

Day 1 images for gels templated with unaligned MAMs for each MAM size (Fig. 6A–C) show maximum cell penetration of ∼40 µm, ∼20 µm, and ∼30 µm into hydrogels templated with 32, 62, and 90 µm MAMs, respectively, with most cells observed at the hydrogel surface. Comparing unaligned gels to gels templated with aligned MAMs at day 1 (Fig. 6G–I), there is a clear difference in overall migration depth in all templated groups and this increase in migration with aligned templated gels was observed across multiple imaged areas and samples (Fig. S5A–F). Quantitative assessment of cell counts after 1 day migration using IMARIS to identify nuclei (Fig. 6M–O), demonstrated maximum depth migration of 125 µm for both 32 and 62 µm templated gels, and up to 225 µm for 90 µm templated gels. However, for all three groups, more than 60% of the observed cells were found between 0–50 µm depth (Fig. S6A, C and E).

Day 3 imaging of hydrogels templated with unaligned MAMs for each MAM size (Fig. 6D–F) shows that Schwann cells still do not migrate much past the surface of hydrogels that do not have aligned pores. In templated but unaligned hydrogels, Schwann cells were observed only in the top 20 µm, 50 µm, and 50 µm of hydrogels templated with an unaligned 32, 62, and 90 µm MAMs, respectively. Similarly to day 1, aligned templated hydrogels in day 3 exhibited a larger migration depth compared to unaligned templated gels (Fig. 6J–L). Cells cultured in aligned templated gels for 3 days migrated up to 175, 225, and 375 µm for gels templated with 32, 62, and 90 µm MAMs, respectively. This maximum migration depth represents increases of 33, 57, and 50% in maximum migration depth compared to day 1 for hydrogels templated with 32, 62 and 90 µm MAMs, respectively. A key observation from the day 3 culture is the increased overall Schwann cell distribution at greater depths across all groups. This demonstrates that the addition of aligned pores facilitates Schwann cell infiltration and suggests that many cells migrate as a front (Fig. S6B, D and F).

Further observations of Schwann cells growing in aligned templated gels (Fig. 6G–L) include apparent differences in cell organization with respect to pore size as they migrate into hydrogels. For example, Schwann cells in the channels of hydrogels templated with 32 µm diameter MAMs seem elongated or stretched into channels at day 1, singly distributed and migrating in sequence while cells in the channels of hydrogels templated using 90 µm diameter MAMs show multiple Schwann cells at the same depth with less elongated shapes. Finally, in hydrogels templated using 62 µm diameter MAMs, Schwann cells extend into channels but groups of cells migrating at the same depth are still observed.

**Fig. 7:**
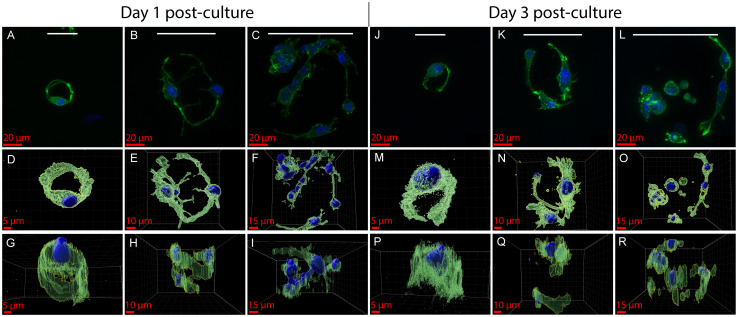
Rat Schwann cell morphology in magnetically templated hydrogels is influenced by channel diameter. Confocal fluorescent images of Schwann cells in hydrogels templated with (A, J) 32, (B, K) 62, and (C, L) 90 µm MAMs at day 1 (A–C) and day 3 (J–L). Top view of reconstructed surfaces using IMARIS in (D) 32, (E) 62, and (F) 90 µm MAM templated hydrogels at day 1 and day 3 (M–O). Side view of reconstructed surfaces using IMARIS in (G) 32, (H) 62, and (I) 90 µm MAM templated hydrogels at day 1 and day 3 (P–R). White bars at the top represent the respective MAM diameter size during templating for comparison of the cell structure.

Given the observations in the migration study, 25× magnification images of rat Schwann cells cultured on templated hydrogels were obtained *via* confocal fluorescence imaging for morphological evaluation of cells in single channels. In templated hydrogels, we observed morphological differences in Schwann cells depending on microchannel diameter (Fig. 7). Maximum intensity projections of *z*-stacks of cells imaged through channels after 1 day of culture (Fig. 7A–C) show that there is a distinct change in organization between Schwann cells infiltrating different sized pores. There is evidence of single Schwann cells wrapped around the microchannels of pores for the hydrogels templated using 32 µm diameter MAMs, forming lumen-like structures (Video S1). As pore size increases, the center of the channel is filled with cells and multiple cells are found at common planes, particularly in the hydrogels templated using 90 µm diameter MAMs (Video S2). The Schwann cells in the hydrogels templated using 62 and 90 µm diameter MAMs also cooperatively wrap around the microchannel walls and cells are found at the center of the channel, thus the cells appear less organized into lumen-like structures (Videos S3 and S4). Side views of cell surfaces in Fig. 7G–I, show multiple cells wrapping around the larger diameter channels through the depth. Cells in hydrogels templated using 32 µm diameter MAMs show a bipolar morphology while cells in the hydrogels templated using the 62 µm and 90 µm diameter MAMs have mixed morphology between unipolar, bipolar, or multipolar. Schwann cells *in vivo* maintain a bipolar phenotype for proper biomechanical function, however, *in vitro*, they exhibit other motile phenotypes. These distinct Schwann cell morphologies have been observed by others in non-patterned 2D and 3D hydrogel cultures with an increasing percentage of bipolar Schwann cells reported in 3D cultures.^70^ Others have shown an increase in the bipolar phenotype as a response to aligned microgrooves.^71^

At day 3, the observed Schwann cells retain their morphological appearance from day 1 and there are multiple cells in the larger diameter microchannels with a mixed morphology whereas single cells with a more bipolar morphology are observed in the hydrogels formed using 32 µm diameter MAMs (Fig. 7J–L). These morphologies were seen throughout different areas of the hydrogels on both day 1 and day 3, suggesting microchannel size affects cell morphology (Fig. S7 and S7). In some cases, in the hydrogels templated using 90 µm diameter MAMs, we observed the formation of clumps of cells in channels in which cells seem to form spheroid like groups (Fig. S8I and Video S5). Further, video representations of the *z*-stacks used to generate these maximum intensity projections and that of other representative channels show that cells tend to migrate sequentially into the channels made with 32 µm diameter MAMs (Video S6), while they tend to migrate in groups or clusters of cells as channel diameter is increased (Video S7).

## 4. Discussion

Incorporating microstructure into hydrogels is considered critical to replicate native tissue topography, porosity, and microenvironment, promoting enhanced cell migration and organization.^72–74^ Porosity of hydrogels plays a key role in the organization of cells, specifically in the context of hierarchically structured tissues like nerve, muscle, and tendon. Further, biomimetic porosity enables the diffusion of nutrients and waste associated with cellular metabolism. In the context of peripheral nerve tissue, micropatterned structures and pores have been shown to play key roles in how cells elongate, orient, migrate, and proliferate.^75^ Micropatterning of hydrogels has been achieved with various techniques like electrospinning, 3D printing, fiber templating, freeze drying, and self-assembly. While these techniques have facilitated micropatterning of structures in biomaterials, are largely confined to 2D patterns, exhibit unorganized porosity, and are often limited to large internal diameters, or limited lengths (see Table 1).

The magnetic templating technique used here allowed for the formation of highly aligned, 3D distributed, cell-scale micropatterned porosities that span millimeter length scales and can be extended to cm length scales.^57^ Consistent with prior work, microfluidic formulation of MAMs enabled the preparation of monodisperse porogens with uniform magnetization.^58^ The observed mismatch between the expected iron oxide loading is attributed to some iron being expelled out of the alginate encapsulation during the acetone wash and the secondary crosslink. During the acetone wash, particles are not yet fully crosslinked and during the secondary crosslink further shrinkage of the alginate capsule is expected. Nano CT of MAM chains and confocal imaging of the resulting microchannels revealed that MAM size plays a role in the physical characteristics of the chains, with smaller MAMs resulting in more chains and microchannels per unit area and volume, whereas larger MAMs led to larger chain length and microchannels, relationships that had not been studied previously. Most chains and microchannels were gap spanning, with high orientational order along the direction of the applied magnetic field. Contrary to other work in 3D micropatterning materials for Schwann cell guidance, we systematically characterized and report pore diameter, pore length, pore areal density, and pore interconnectivity. When compared to the commonly described physical properties of scaffolds, our approach achieves similar channel lengths with cell-scale diameters while offering higher areal densities than most existing methods. Processes like alginate capillary self-assembly do achieve higher areal density of pores, but this method relies on diffusion to form capillaries, which limits achievable channel lengths and the time to achieve them.^47^ Although not all prior literature explicitly reports areal densities, several do mention the number of total channels which typically range from 1–12 total channels.^32,37,38,52,76^ As in prior work, qualitative clearance of MAMs was achieved in fewer than 7 days, based on changes in hydrogel color. However, in this work we tracked iron removal quantitatively utilizing MPI and showed two burst releases, one occurring upon immediate immersion on the EDTA likely due to surface MAM diffusion and a second occurring at day 3, which could result from slower MAM degradation. As with prior findings, indentation characterization of templated gels demonstrated that magnetic templating imparts anisotropic mechanical stiffness. Although MAM diameter influenced microchannel diameter, length, volume and areal density, and interconnectivity, it played a minor role in the resulting mechanical properties. These observations illustrate the potential of tuning microstructure of magnetically templated hydrogels while maintaining mechanical properties that mimic those of nerve tissue.

Volume and areal density measurements of channels from confocal images align with chain numbers obtained from nano CT, showing a consistent trend where smaller porogens result in higher areal and volume channel densities. Furthermore, lower median channel lengths estimated from confocal microscopy are attributed to the lower contrast generated by fluorescence imaging compared to high nano CT contrast generated from the iron in MAMs. Specifically, reduced fluorophore presence was observed towards the edges of the gel likely due to passive diffusion into the surrounding media. Differences in median lengths between nano CT and confocal were of 25, 24, and 1% for gels templated with 32, 62, and 90 µm MAMs, respectively. Regardless, confocal assessment of channel physical length showed the same trend of increasing median length with increasing MAM size as observed in nano CT and confirms the retainment of microchannels upon MAM removal.

*In vitro* evaluation of rat Schwann cell migration into hydrogels showed that aligned microporosity, obtained through magnetic templating, enhanced migration of cells compared to hydrogel controls with unaligned pores. We observed both maximum migration depth and the fraction of cells penetrating deeper into the hydrogel increased with increasing microchannel diameters in both days 1 and 3. While higher number of cells migrating in larger pores is consistent with other reports,^35^ relative cell distribution as a function of hydrogel depth has not been widely reported. Additionally, we observed differences in Schwann cell morphology and organization as a function of pore diameter, with smaller pores leading to predominant spindle or bipolar morphologies, whereas larger pores led to a mixed population of cellular morphologies. This variation in Schwann cell morphology has been previously observed in the context of 2D *vs.* 3D peripheral nerve tissue cultures of Schwann cells, and suggests that the fraction of polarized cells increases in 3D hydrogels.^70^ However, this has not been widely studied as a function of 3D hydrogel characteristic pore size, and our observations demonstrate pore diameter is an important contributor to cell organization in these hydrogels. These observations suggest that achieving a balance between enhancing cell migration density and retaining relevant cues for morphologically appropriate organization is a key factor to consider when designing hydrogels for peripheral nerve repair.

Although this study demonstrates the potential of magnetically templated hydrogels with size-tunable and highly aligned channels that enhance and influence cell migration and morphology, further work is needed. In the context of the porogens studied, smaller MAMs could enable exploration of the role of even smaller porosities, which are more biologically relevant as channels that remain after decellularization of peripheral nerve tissue possess diameters between ∼10–20 µm.^41^ Implementation of stronger magnetic fields could also enable improved chain length and alignment to form more hydrogel spanning channels through which cells can fully migrate instead of relying on their ability to degrade the hydrogel matrix. Areal channel density is also an important factor. Axon areal density in rat sciatic nerve is reported in the range of 1000–8000 mm^−2^.^77,78^ The channel densities observed in these magnetically templated hydrogels are much lower. This limitation is of specific interest and parameters like MAM volume fraction should be studied in the context of smaller MAMs. Further assessment of the role of aligned channel microstructures on cell secretome behavior could greatly expand the potential of these templated hydrogels. Evaluating expression and deposition of key proteins involved in nerve repair or cell phenotype would enable assessment of cell function (*i.e.* myelinating *vs.* repair Schwann cell). In addition, growth factor gradients through hydrogels could be incorporated to evaluate their role on migration in a potentially disease-relevant manner. While these hydrogels are composed of biologically active polymers (collagen and hyaluronic acid) further functionalization with peptides like YIGSIR or IKVAV, specifically if done to the inside walls of the aligned channels could greatly enhance cell migration and organization thereby adding further tunability to the biomaterial to direct cellular response.

We believe that with further work, magnetically templated hydrogels could lead to exciting applications in tissue engineering and regenerative medicine. First, our approach to micropatterning porosities allows for cell-scale (<50 µm) highly aligned, homogeneously distributed mm–cm scale porosities which are unavailable with other techniques. Further exploration using smaller MAMs could lead to better probing of cell response specifically *in vivo* or multi-cellular cultures *in vitro*. For example, at a 2.2% v/v porogen loading (used in this work), areal density of chains/channels seems to be proportional to the square of the MAM diameter. If we assume this relation holds for a 10 µm MAM we would expect to achieve areal densities between 200–300 chains per mm^2^ which would greatly enhance the hydrogel's ability to mimic the pore diameter and the high areal densities found in peripheral nerve. Further, increasing the loading of MAMs will also increase the number of particles and the achievable areal densities. Due to our hydrogel chemistry relying on photo-initiation and MAMs being opaque because of iron loading, there is an upper limit to the volume fraction of MAMs that can be incorporated without hindering UV crosslinking efficiency. Thus, future work in expanding the magnetic templating process to thermally crosslinked polymers in which the number of particles can be greater without interfering with hydrogel crosslinking would open new avenues for the formation of micropatterned hydrogels with higher areal density of channels.

## 5. Conclusions

Microscale patterning of hydrogels with aligned porosity is considered critical in the rational design of cell-instructive biomaterials. Here we formulated natural polymer hydrogels with tunable and highly aligned micropores by using a sacrificial magnetic alginate microparticle porogen. We comprehensively characterized the physical architecture imparted in the templating process and demonstrated that MAM diameter plays a role in the areal density, diameter, and length of the patterned structures. MAM clearance rate is not affected by MAM diameter, and mechanical characterization confirmed anisotropic properties that were weakly dependent on the diameter of the MAMs used to prepare the hydrogels. We confirmed that MAM clearance resulted in channels with length, diameter, areal density, volume density, and branching distributions closely matching those of the chains formed in the hydrogels during the magnetic templating process before clearance. Lastly, we studied the role of pore diameter and length on Schwann cell morphology and migration depth and demonstrate that smaller pores lead to bipolar Schwann cell morphologies, while larger diameter channels led to deeper Schwann cell penetration into the hydrogels. Future work aims at templating smaller pores for peripheral nerve engineering and evaluating multicellular cultures.

## Author contributions

The manuscript was written through contributions of all authors. All authors have given approval to the final version of the manuscript.

Victor Rivera-Llabres: conceptualization, methodology, validation, formal analysis, investigation, data curation, writing – original draft, visualization. Zoe A. Fields: methodology, formal analysis, investigation, writing – original draft. Hayden J. Good: methodology, formal analysis, investigation, writing – original draft. Elizabeth L. Aikman: methodology, investigation. Stephanie Manrique: investigation. Adeline O. Schmidt: investigation. Eleana Manousiouthakis: methodology. Whtiney L. Stoppel: resources, writing – review and editing, supervision, funding acquisition, Christine E. Schmidt: resources, writing – review and editing, supervision, funding acquisition. Carlos M. Rinaldi-Ramos: conceptualization, resources, writing – review & editing, supervision, funding acquisition.

## Conflicts of interest

There are no conflicts to declare.

## Abbreviations

MAMs: Magnetic alginate microparticles
MPI: Magnetic particle imaging

## Supplementary Material

BM-014-D5BM01573A-s001

BM-014-D5BM01573A-s002

BM-014-D5BM01573A-s003

BM-014-D5BM01573A-s004

BM-014-D5BM01573A-s005

BM-014-D5BM01573A-s006

BM-014-D5BM01573A-s007

BM-014-D5BM01573A-s008

BM-014-D5BM01573A-s009

## Data Availability

Data collected for this article including description of data have been included as supplementary information (SI). Supplementary information is available. See DOI: https://doi.org/10.1039/d5bm01573a.
