# Supplementary material for: Tuning hydrogel properties and Schwann cell behavior through microchannel size control in magnetically templated hydrogels
Source: Biomater Sci. 2026 Apr 30;14(11):2995–3012. doi: 10.1039/d5bm01573a (PMC13130880; doi:10.1039/d5bm01573a)
Supplement: BM-014-D5BM01573A-s002 [file BM-014-D5BM01573A-s002.pdf]

Supplementary Information (SI) for Biomaterials Science.
This journal is © The Royal Society of Chemistry 2026

Tuning hydrogel properties and Schwann cell behavior through microchannel size control in magnetically templated hydrogels

Victor G. Rivera-Llabres[1], Zoe A. Fields[1], Hayden J. Good[1], Elizabeth L. Aikman[1], Stephanie Manrique[2], Adeline Schmidt[1], Eleana Manousiouthakis[3], Whitney L. Stoppel[1,3], Christine E. Schmidt[1,3], and Carlos M. Rinaldi-Ramos[1,3,]*

[1]Department of Chemical Engineering, University of Florida, Gainesville, FL 32611, USA

[2]Department of Agricultural and Biological Engineering, University of Florida, Gainesville, FL 32611, USA

[3]J. Crayton Pruitt Department of Biomedical Engineering, University of Florida, Gainesville, FL 32611, USA

*Corresponding author.

Email address: carlos.rinaldi@ufl.edu (C.M. Rinaldi-Ramos)

Keywords: tissue engineering, peripheral nerve, Schwann cell, cell morphology, hydrogel, pores, porogen, magnetic microparticles, micropatterning

Supporting information

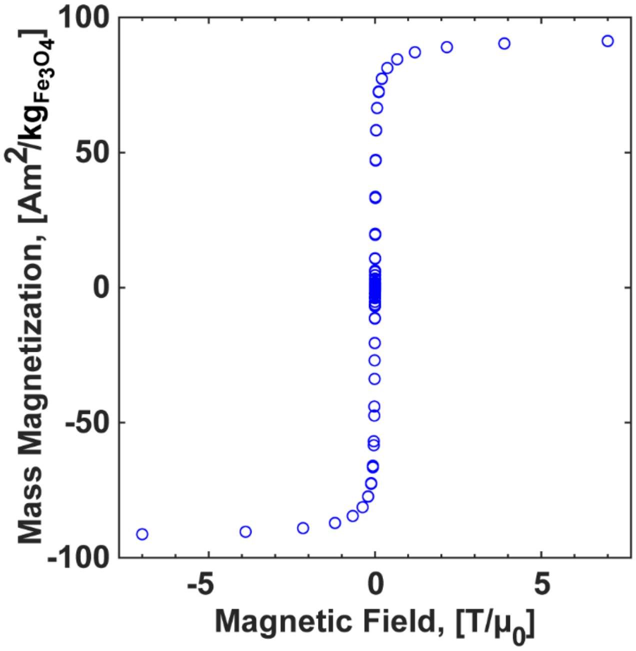

Figure S1. Liquid sample magnetization curves for the free iron oxide nanoparticles used to formulate magnetic alginate microparticles. Magnetization is normalized by iron oxide mass.

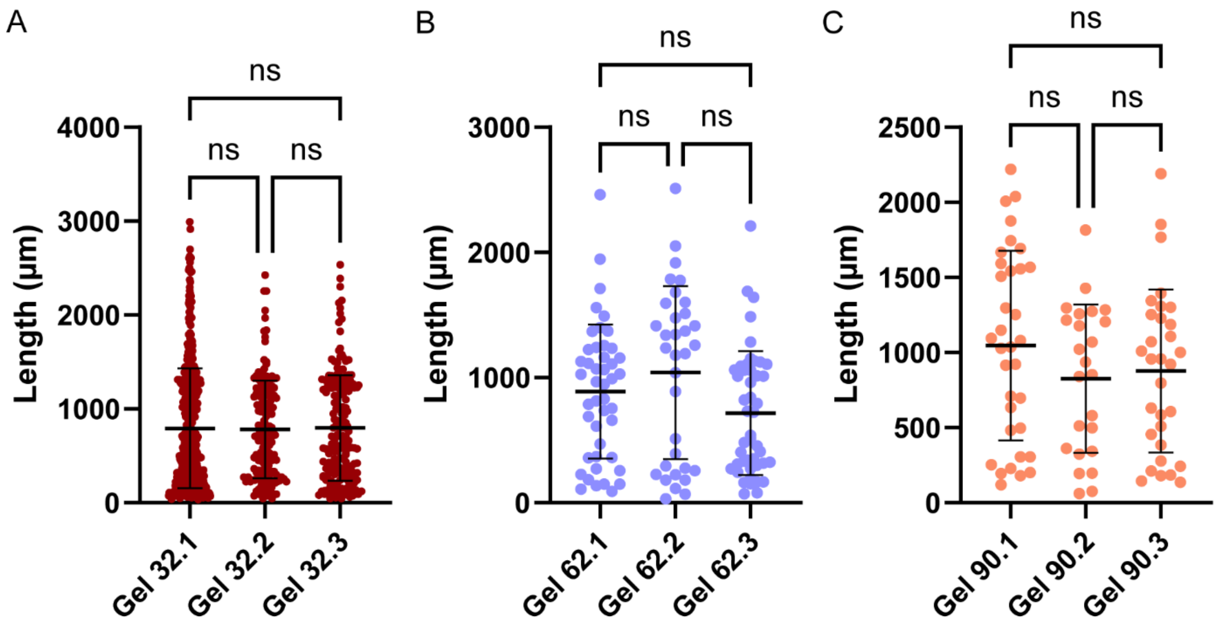

Figure S2. Channel length distributions are consistent across multiple batches of hydrogels templated with the same MAM diameter. Three hydrogels were prepared, cleared, and analyzed for channel length distribution using confocal microscopy for each group. (A) Channel length distribution for hydrogels templated using 32 µm diameter MAMs. (B) Channel length distribution for hydrogels templated using 62 µm diameter MAMs. (C) Channel length distribution for hydrogels templated using 90 µm diameter MAMs. A Brown-Forsythe one-way ANOVA with Tukey's post-hoc analysis was performed to evaluate for statistical significance.

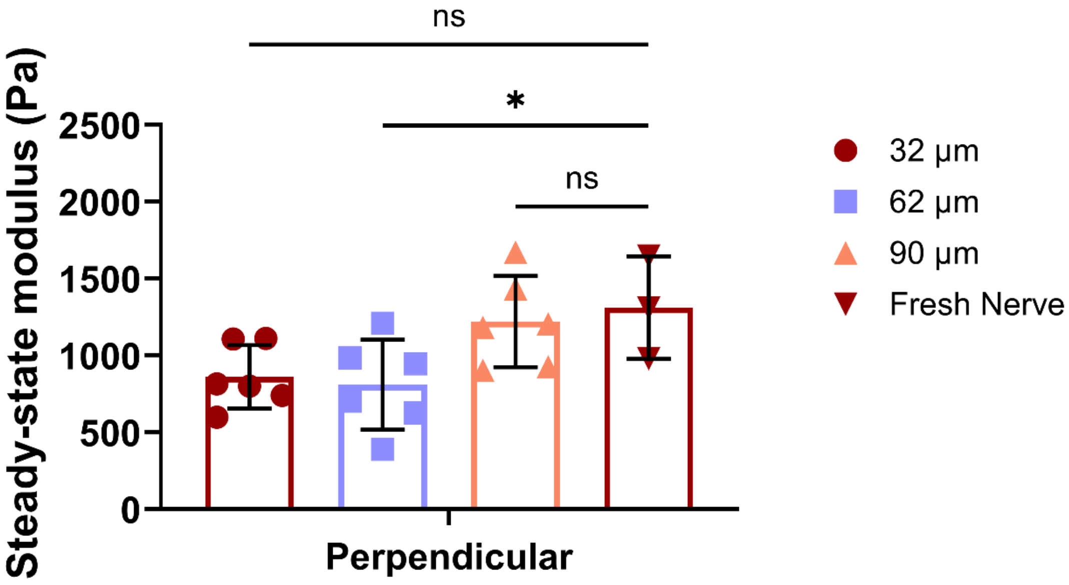

Figure S3. Magnetically templated hydrogels with channels perpendicular to indentation tip possessed mechanical properties comparable to those of freshly explanted rat nerve.

Statistical comparisons were obtained by performing a 1-way ANOVA and Tukey's post-hoc comparisons (n = 6).

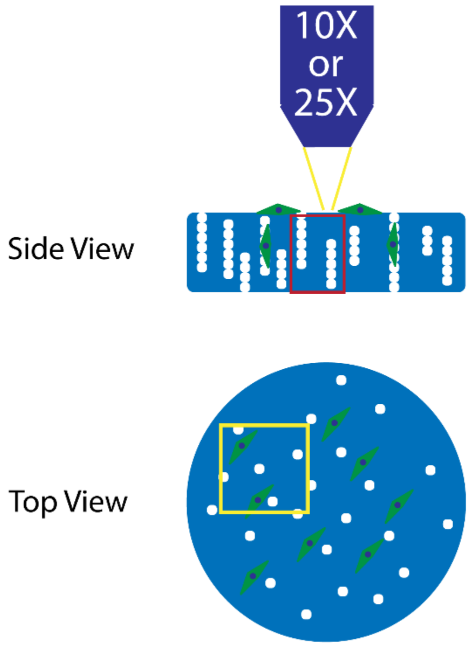

Figure S4. Rat Schwann cell seeding and imaging direction using a confocal microscope. The yellow square indicates the imaging area which is dependent on objective magnification. The red rectangle denotes the projected side views shown in Figure 6. Multiple images were acquired at different heights of the yellow area in the top view to look through the depth of the hydrogel and form a 3D image. The yellow square denotes the imaging used for the images in Figure 7.

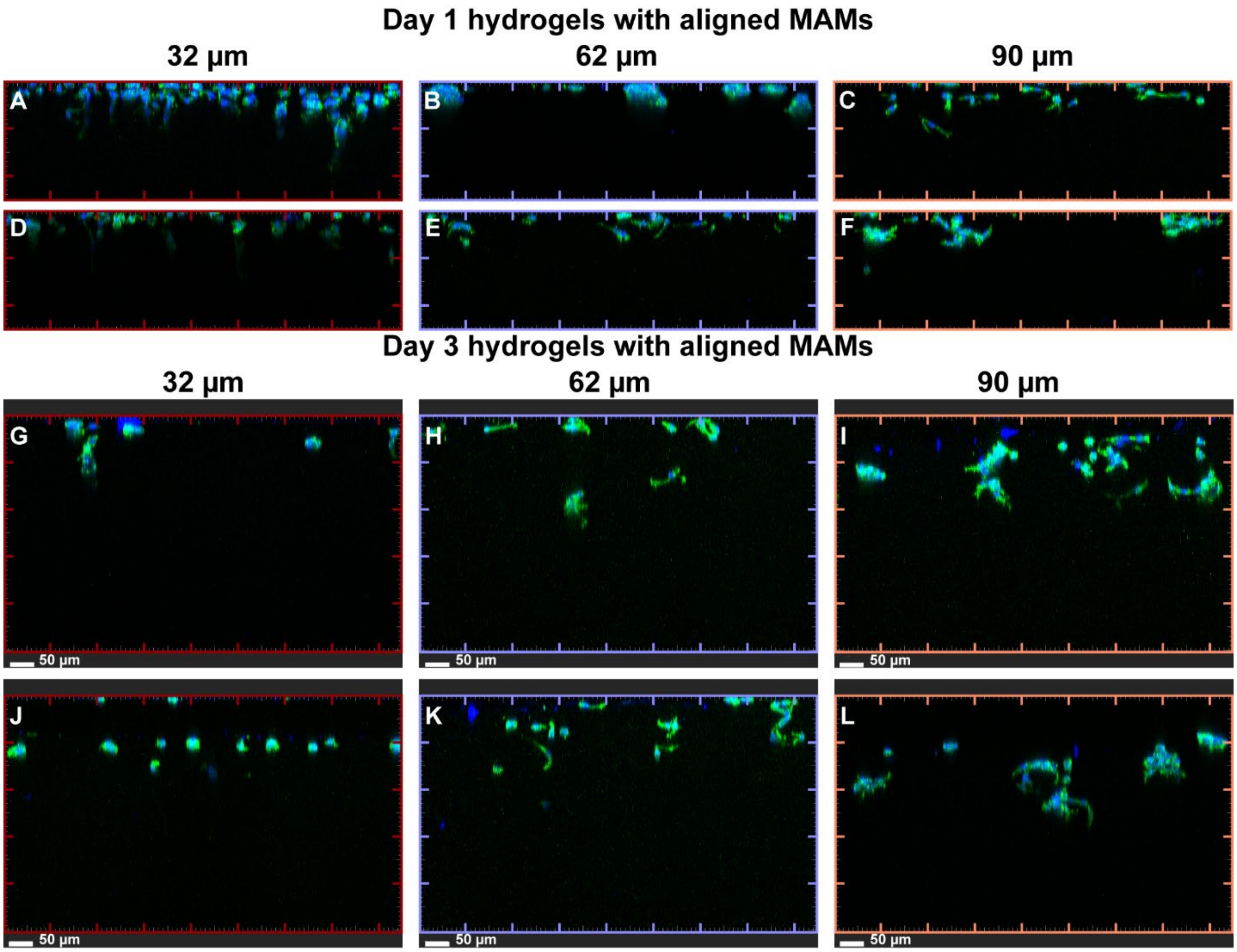

Figure S5. Additional observations of rat SC migration into magnetically templated hydrogels. Representative maximum intensity projections of size views of z-stacks obta
ined at 10X magnification for day 1 and 3 post migration. Migrating rat Schwann cells seeded in hydrogels with aligned (A,D,G,J) 32 µm, (B,E,H,K) 62 µm, and (C.F,I,L) 90 µm MAMs imaged at (A-F) day 1 and (G-L) day 3 after seeding. Major tick in the grid represents 100 µm.

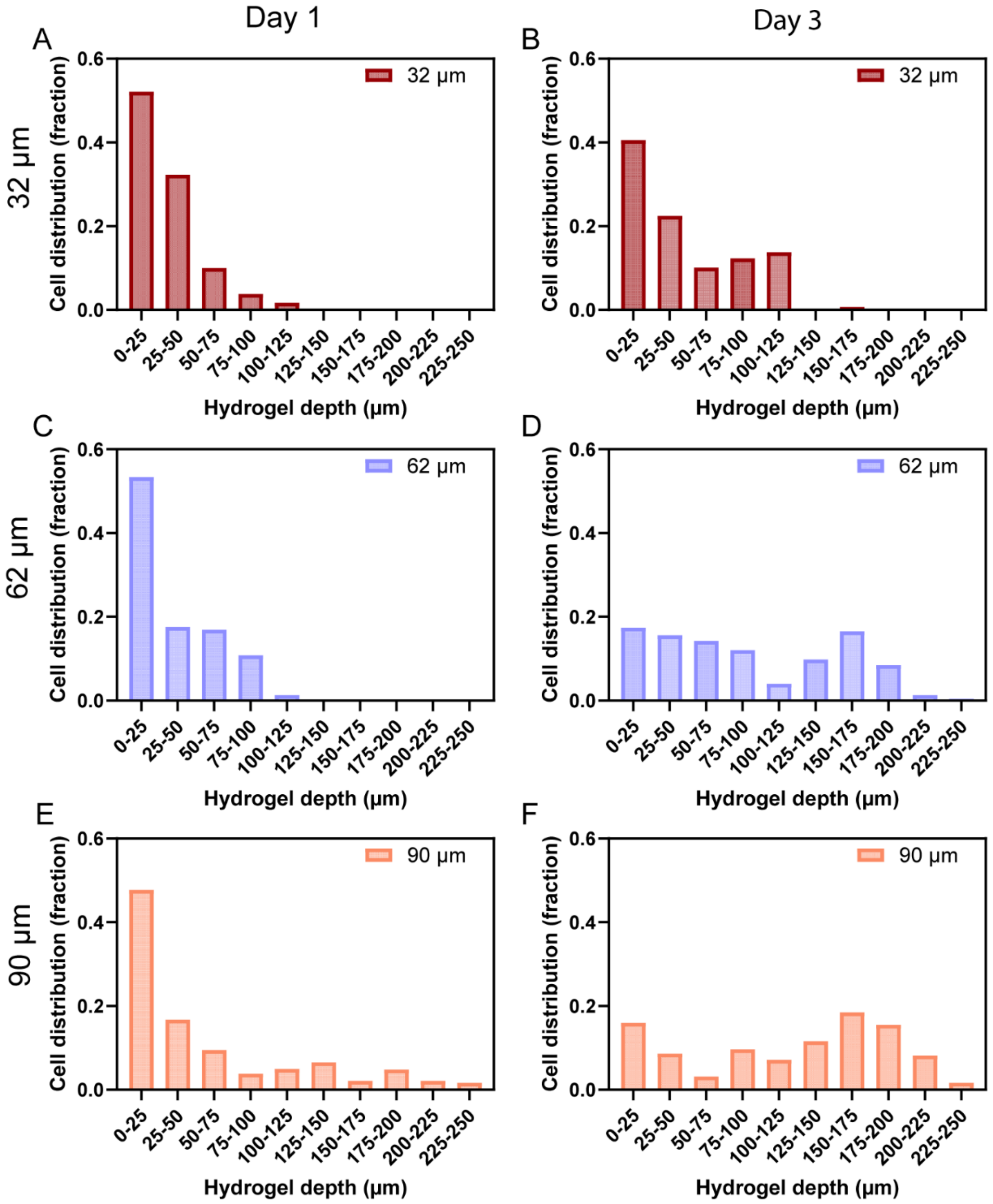

Figure S6. Average cell probability distribution fractions for cells cultures in magnetically templated hydrogels obtained using magnetic alginate microparticles of different diameters. Cell distribution after (A,C,E) 1 and (B,D,F) 3 days of culture in hydrogels templated with (A,B) 32 µm, (C,D) 62 µm, and (E,F) 90 µm MAMs.

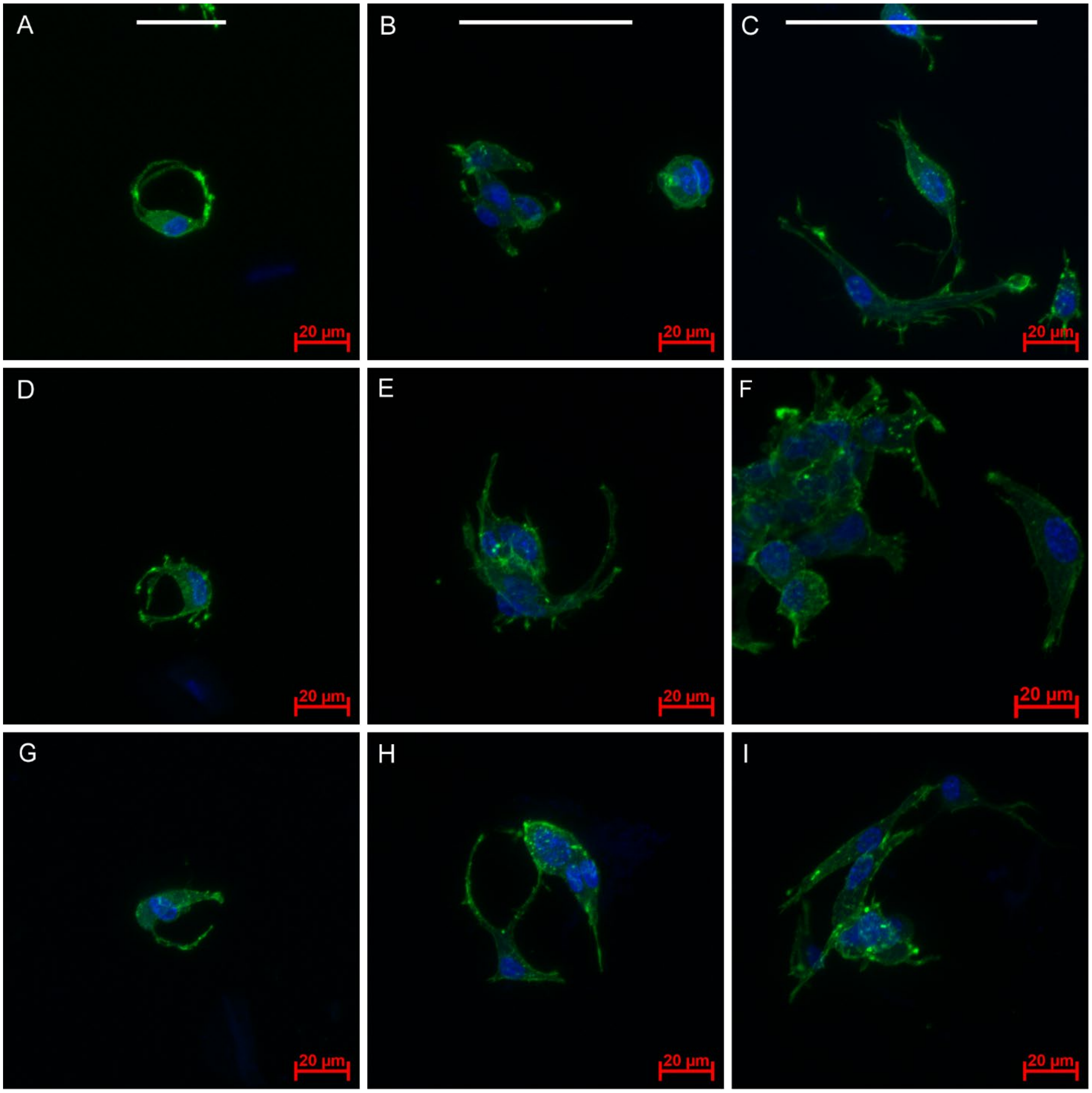

Figure S7. Additional representative images of Schwann cell morphology in different locations of the magnetically templated hydrogels after 1 day in culture for hydrogels obtained using 32 µm (A,D,G), 62 µm (B,E,H), and 90 µm (C,F,I) MAMs. The white scale bar at top of the figure in panel A, B, and C is to scale with the diameter of the MAMs used in the templating process.

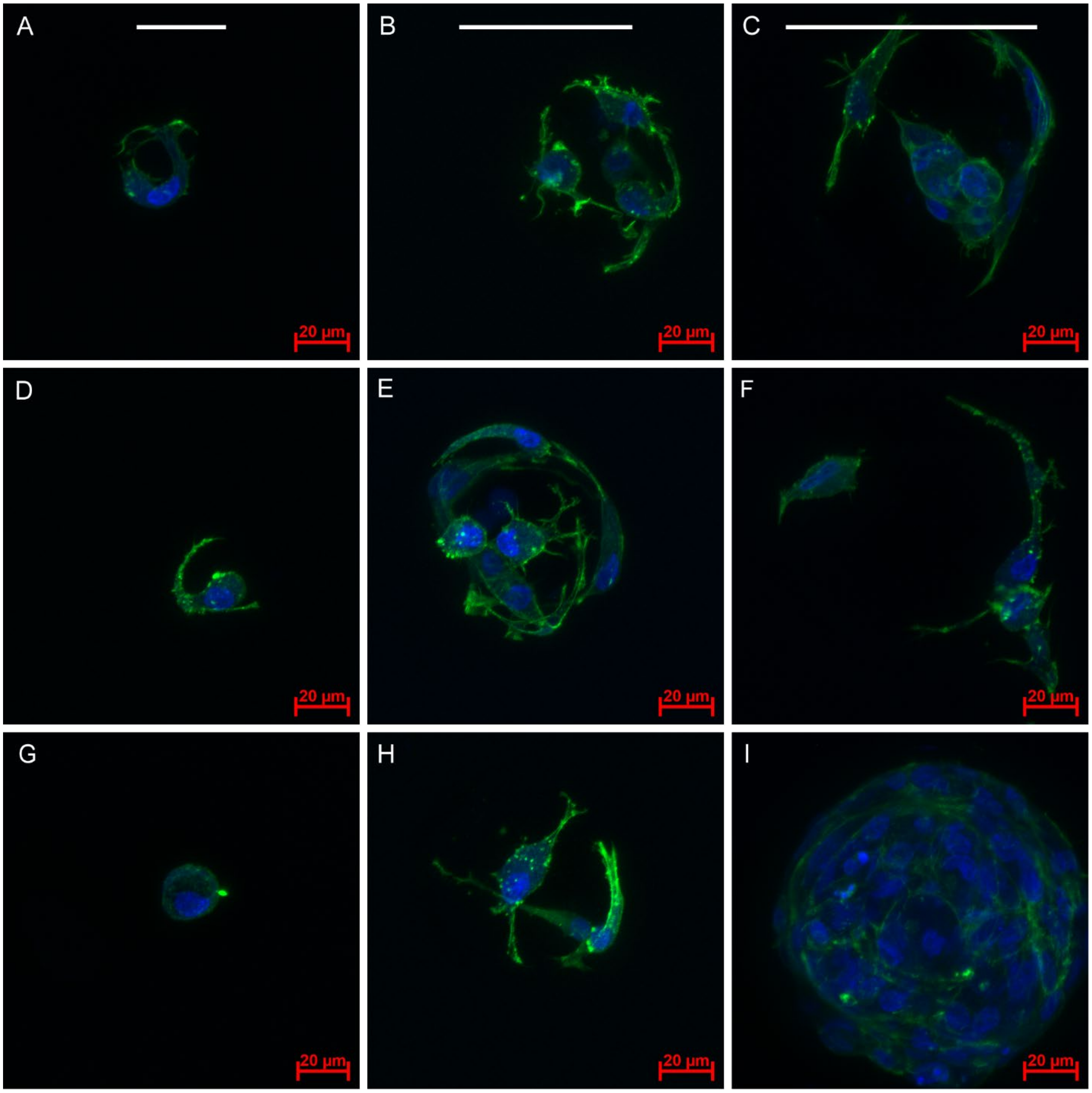

Figure S8. Additional representative images of Schwann cell morphology in different locations of the magnetically templated hydrogels after 3 days in culture for hydrogels obtained using 32 µm (A,D,G), 62 µm (B,E,H), and 90 µm (C,F,I) MAMs. The white scale bar at top of the figure in panel A, B, and C is to scale with the diameter of the MAMs used in the templating process.
